# Mismatch repair deficiency in metastatic prostate cancer: Response to PD-1 blockade and standard therapies

**DOI:** 10.1371/journal.pone.0233260

**Published:** 2020-05-26

**Authors:** Laura S. Graham, Bruce Montgomery, Heather H. Cheng, Evan Y. Yu, Peter S. Nelson, Colin Pritchard, Stephanie Erickson, Ajjai Alva, Michael T. Schweizer

**Affiliations:** 1 Division of Oncology, Department of Medicine, University of Washington, Seattle, WA, United States of America; 2 Clinical Research Division, Fred Hutchinson Cancer Research Center, Seattle, WA, United States of America; 3 VA Puget Sound Health Care System, Seattle, WA, United States of America; 4 Division of Human Biology, Fred Hutchinson Cancer Research Center, Seattle, WA, United States of America; 5 Department of Lab Medicine, University of Washington, Seattle, WA, United States of America; 6 Division of Hematology-Oncology, Department of Medicine, University of Michigan, Ann Arbor, Michigan, United States of America; University of Minnesota Twin Cities, UNITED STATES

## Abstract

**Background:**

While response rates to anti-PD1 therapy are low in unselected metastatic castration resistant prostate cancer (mCRPC) patients, those with inactivating mutations in mismatch repair (MMR) genes (i.e. MMR deficiency; MMRd) or microsatellite instability (MSI) are thought likely to respond favorably. To date, there is limited published data on this biologically distinct and clinically relevant subgroup’s natural history and response to treatment.

**Methods:**

We retrospectively identified patients at two academic institutions who had MMRd/MSI-high metastatic prostate cancer (PC). Clinical and pathologic characteristics at the time of diagnosis as well as response to standard therapies and immune checkpoint therapy were abstracted. Descriptive statistics, including PSA50 response (≥50% decline in PSA from baseline) and clinical/radiographic progression free survival (PFS), are reported.

**Results:**

27 men with MMRd and/or MSI-high metastatic PC were identified. 13 (48%) men had M1 disease at diagnosis and 19 of 24 (79%) men that underwent prostate biopsy had a Gleason score ≥8. Median overall survival from time of metastasis was not reached (95% CI: 33.6-NR mos) after a median follow up of 33.6 mos (95% CI: 23.8–50.5 mos). Seventeen men received pembrolizumab, of which 15 had PSA response data available. PSA50 responses to pembrolizumab occurred in 8 (53%) men. Median PFS was not reached (95% CI: 1.87-NR mos) and the estimated PFS at 6 months was 64.1% (95% CI: 33.7%-83.4%). Of those who achieved a PSA50 response, 7 (87.5%) remain on treatment without evidence of progression at a median follow up of 12 months (range 3–20 months).

**Conclusions:**

MMRd PC is associated with high Gleason score and advanced disease at presentation. Response rates to standard therapies are comparable to those reported in unselected patients and response rate to checkpoint blockade is high. Our study is limited by small sample size, and more research is needed to identify additional factors that may predict response to immunotherapy.

## Introduction

Early studies testing immune checkpoint inhibitors in unselected men with advanced prostate cancer have demonstrated minimal success to date. In two placebo-controlled Phase III studies, the CTLA4 inhibitor ipilimumab failed to demonstrate improvements in overall survival (OS) in men with metastatic castration-resistant prostate cancer (mCRPC) [1, 2]. Similarly, PD1-pathway blockade with pembrolizumab in docetaxel-refractory mCRPC patients has demonstrated low response rates (3–5%) [3]. These studies have diminished enthusiasm for checkpoint inhibitors as monotherapies in unselected mCRPC patients. Combination therapy appears to have higher response rates but with added toxicity. Preliminary results from a phase II study investigating the combination of the CTLA-4 inhibitor ipilimumab with the PD-1 inhibitor nivolumab showed a 25% response rate in men whose PC had progressed after second-generation hormonal therapy, and a 10% response rate in men whose PC had progressed after hormonal therapy and chemotherapy [4]. These modestly higher response rates came with greater toxicity, with 40–50% of men reporting grade 3–5 adverse events and 33–35% coming off study due to adverse events [4]. Several studies are ongoing to evaluate novel combination immunotherapy approaches and/or to evaluate checkpoint inhibition in patients whose tumors display candidate molecular features predicting for response.

Loss of function alterations in mismatch repair (MMR) genes (i.e. *MLH1*, *MSH2*, *MSH6* and *PMS2*) define a subset of patients with high potential for responses to immune checkpoint blockade. The MMR genes are involved in maintaining genomic fidelity during cellular replication, and when MMR deficiency (MMRd) occurs through mutational or epigenetic events, tumor cells can demonstrate evidence of somatic hypermutation–often reflected as microsatellite instability (MSI). Hypermutation, in turn, is associated with higher expression of tumor neoantigens which facilitates immune recognition [5]. These observations have served as the biologic foundation for testing pembrolizumab (anti-PD1) in MMRd/MSI-high cancers, and led to the FDA approval of pembrolizumab for the treatment of microsatellite instability-high (MSI-H) or MMRd solid tumor malignancies, regardless of tissue of origin. Since this approval was tumor type agnostic, little was known about the specific response in prostate cancer. More recently, Abida, *et al*. described 32 patients with MSI-H/MMRd prostate cancer, 11 of whom received pembrolizumab with a PSA50 response rate (≥50% decline in PSA from baseline) of 54.5% [6]. Antonarakis, *et al*. described 11 patients with MMR-mutated advanced prostate cancer, 4 of whom received anti-PD-1 therapy (either pembrolizumab or nivolumab) and 2 (50%) achieved a PSA50 response [7].

Given the relatively low (3–12%) prevalence of MMRd in prostate cancer, the clinical behavior of this biologic subgroup is incompletely defined [6, 8–11]. The knowledge of the clinical and pathologic characteristics of men with MMRd/MSI-H prostate cancer is still evolving and could have significant therapeutic implications. Here we report a cohort of men with either germline or somatic mutations in mismatch repair genes, or evidence of microsatellite instability, and specifically focus on treatment response to not only immune checkpoint blockade, but also standard therapies.

## Methods

We identified consecutive patients at two academic institutions who had metastatic prostate cancer and either a pathogenic inactivating mutation in one of the MMR genes (i.e. *MLH1*, *MSH2*, *MSH6*, or *PMS2*) or evidence of microsatellite instability on next-generation sequencing. A variety of clinical grade sequencing assays were used to assess MMR and/or MSI status, including somatic tumor assays: UW-OncoPlex, MiOncoSeq, whole exome sequencing (WES), and the germline assays: Color Genomics and Invitae Genetics [9, 12, 13]. Only MMR gene alterations deemed likely pathogenic were used for determining inclusion in this study. Cases with monoallelic MMR gene alterations were included given that we could not rule out epigenetic silencing or cryptic genomic events affecting the other allele [14]. This study was approved by the institutional review boards of University of Washington and University of Michigan. The requirement for informed consent was waived. Data was collected by retrospective chart review and was not fully anonymized.

Clinical and pathologic characteristics at the time of diagnosis as well as response to standard therapies and immune checkpoint therapy were abstracted retrospectively. Clinical efficacy variables included PSA change from baseline and clinical and/or radiographic progression. Analyses were descriptive in nature and included PSA50 response (≥50% reduction in PSA from baseline), with minimum time to assess PSA response of 12 weeks, as well as clinical or radiographic progression free survival (PFS). Kaplan-Meier method was used to estimate survival endpoints and results are reported with 95% CI. All analyses were conducted using the statistical software STATA or Prism.

## Results

### Patients

We identified 27 men with MSI-high or MMRd metastatic prostate cancer. Eleven were from University of Michigan and 16 were from University of Washington. Their baseline characteristics are reported in Table 1. Full genetic and pathologic characteristics for individual patients are reported in the S1 Table. Thirteen (48%) had *de novo* metastatic disease at the time of diagnosis and 19 of 24 (79%) men that underwent prostate biopsy had Gleason score 8–10 disease and 8 of 24 (33%) had evidence of ductal histology. The most commonly mutated gene was *MSH2* (20, 74%). One patient did not have a detectable MMR gene mutation but their tumor had evidence of microsatellite instability. All patients received standard medical/surgical castration as initial therapy for metastatic prostate cancer. Two men received abiraterone for hormone sensitive prostate cancer (HSPC) and 5 men received docetaxel for HSPC. Median time to CRPC on first-line ADT was 14.2 months (95% CI: 8.03–32.6 mos). With a median follow up of 33.6 mos (95% CI: 23.8–50.5 mos), the median overall survival from time of metastasis was not reached (95% CI: 33.6-NR mos).

**Table 1:** Baseline characteristics.

Median age at Diagnosis, year (range)	65 (52–90)
Caucasian race-, N (%)	27 (100)
Gleason score, N (%)	
7	5 (19)
8	2 (7)
9	17 (63)
Unknown	3 (11)
Presence of ductal/intraductal histology, N (%)	8 (30)
Presented with metastatic disease at diagnosis, N (%)	13 (48)
Affected MMR gene	
MSH2 mutation (%)	20 (74)
MSH6 mutation (%)	5 (19)
PMS2 mutation (%)	2 (7)
MLH1 mutation (%)	1 (4)
Prior systemic therapies	
ADT, N (%)	27 (100)
Abiraterone, N (%)	21 (78)
Enzalutamide, N (%)	11 (41)
Docetaxel, N (%)	16 (59)
Cabazitaxel, N (%)	6 (22)
Sipeleucel-T, N (%)	1 (4)
Radium-223,N (%)	2 (7)
Pembrolizumab, N (%)	17 (63)

MMR, mismatch repair; ADT, androgen deprivation therapy

### Response to docetaxel

**Fig 1:**
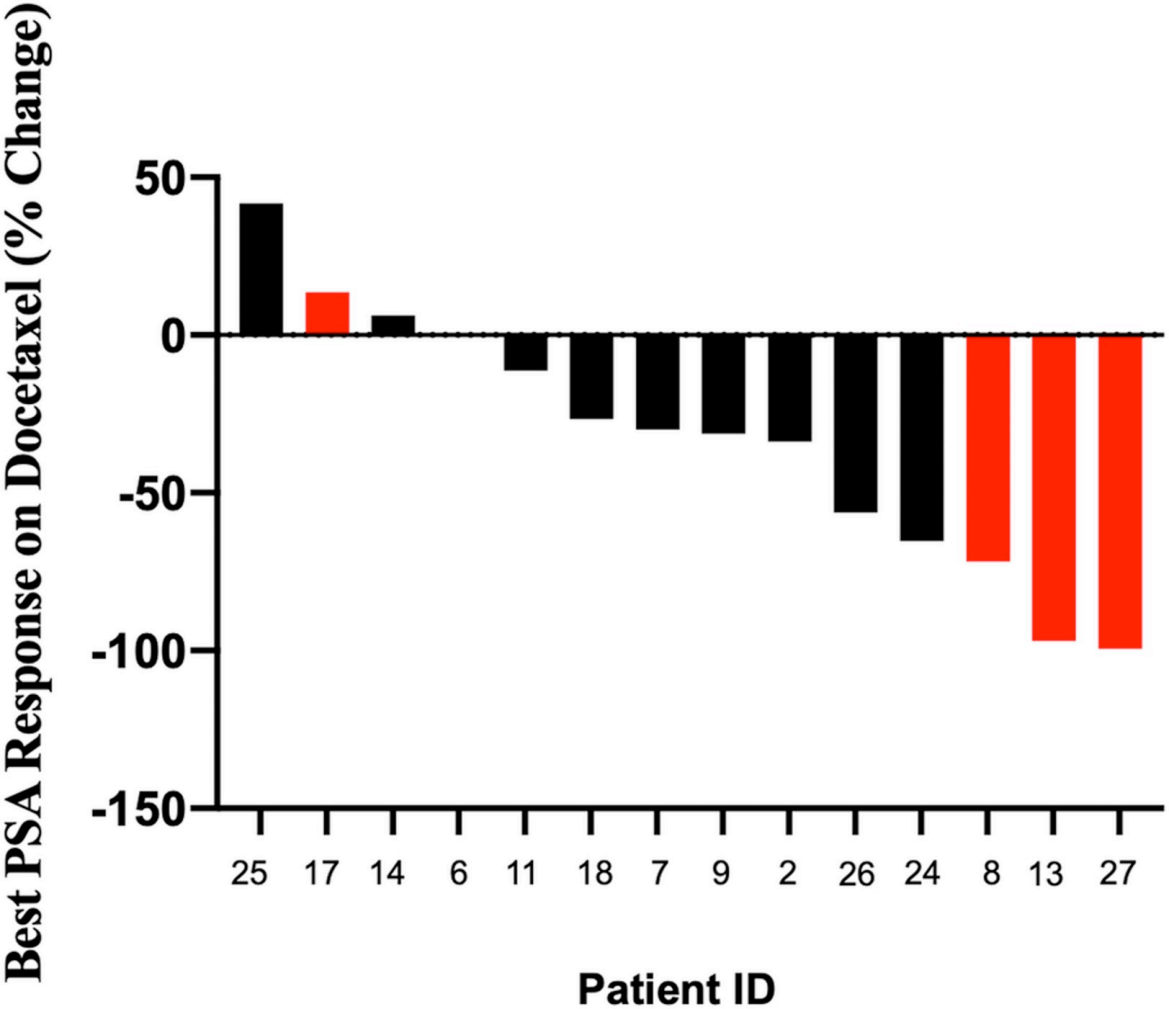
Best PSA response on docetaxel in men with MMRd/MSI-H PC. Best % PSA change from baseline following treatment with docetaxel. Red = docetaxel used for hormone-sensitive disease. Black = docetaxel used for castration-resistant disease.

Sixteen men received docetaxel, 5 in the hormone sensitive setting and 11 in the castration-resistant setting. Two men did not have PSA data available–one due to rapid progression and transition to comfort care, the other because he received docetaxel outside of our systems. The percent of men who achieved a PSA50 response are shown in Fig 1. Three (60%) men who received docetaxel in the hormone-sensitive setting had a PSA50 response, compared to 2 (22%) patients who received docetaxel in the CRPC setting. Median PFS in the CRPC setting was 3.8 months (95% CI: 0.36-NR mos) (Table 2).

**Table 2:** Response to standard therapy in men with MMRd/MSI-H PC.

	N	PSA50 Response, N (%)	Median PFS, Months (95% CI)
**Abi for HSPC**	2	2 (100)	N/A
**Abi/Enza 1**^**st**^ **line for CRPC**	16	10 (62.5)	8.56 (3.73–9.51)
**Abi/Enza 2**^**nd**^ **line for CRPC**	9	3 (33)	4.59 (1.83–11.05)
**Docetaxel for CRPC**	9	2 (22)	3.8 (0.36-NR)

Abi, abiraterone; Enza, enzalutamide

### Response to AR-signaling inhibitors

**Fig 2:**
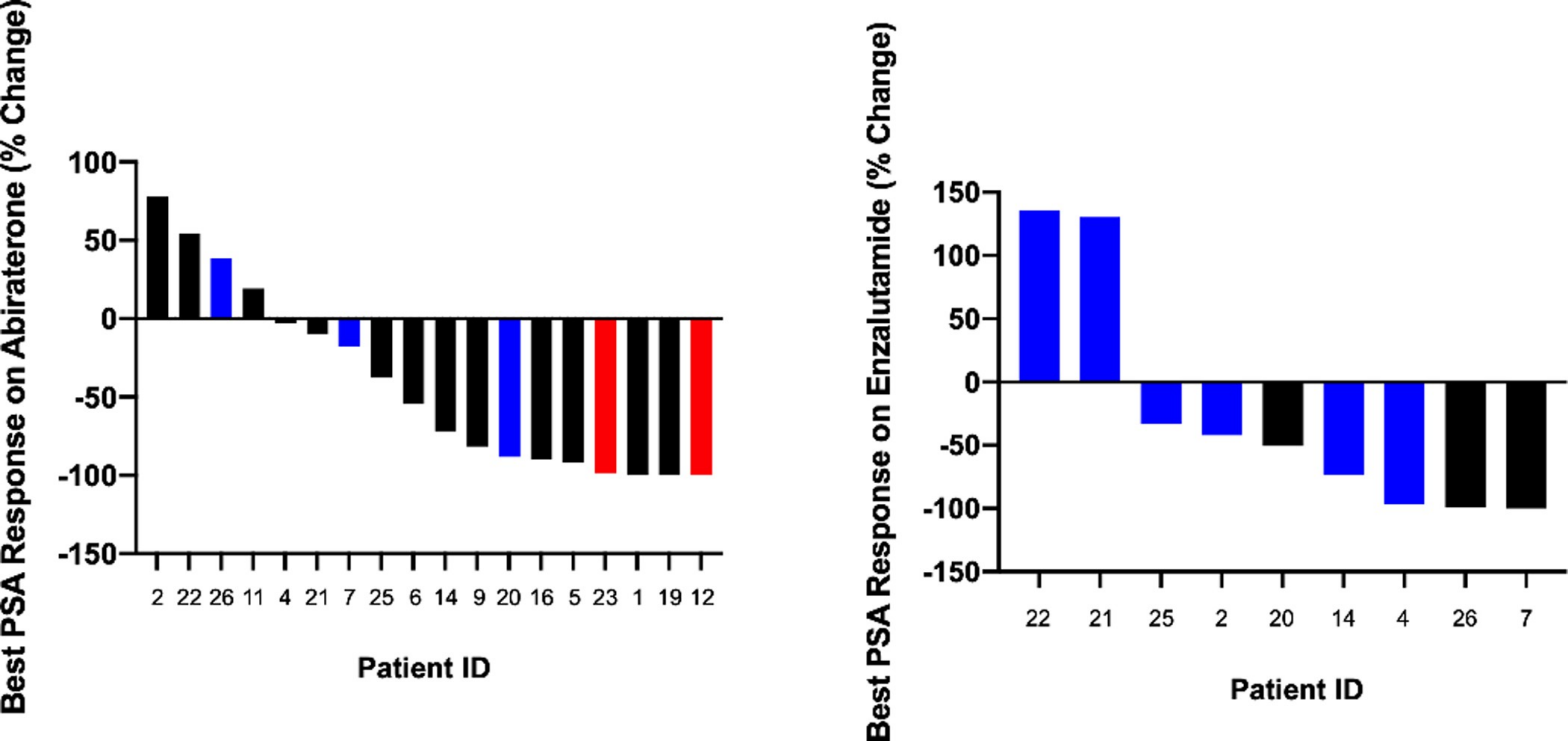
Best PSA response on second generation hormonal agents in men with MMRd/MSI-H PC. Best % PSA change from baseline following treatment with abiraterone (A) and enzalutamide (B). Red = agent used for hormone-sensitive disease. Black = agent used as 1^st^ line treatment for mCRPC. Blue = agent used as 2^nd^ line agent for mCRPC.

Responses to second-generation hormonal agents were also examined. Twenty-one patients received abiraterone acetate: 2 (9.5%) in the hormone sensitive setting and 19 (90.5%) for castration-resistant disease. Both patients (100%) treated with abiraterone for HSPC had a PSA50 response. Sixteen patients who had abiraterone for mCRPC had PSA data available. PSA50 responses were observed in 7 (54%) and 1 (33%) of men who received abiraterone as first-line or second-line therapy after enzalutamide for mCRPC, respectively (Fig 2A).

Twelve men received enzalutamide: 5 received it as first-line therapy for mCRPC and 7 as second-line therapy following abiraterone given for mCRPC. Nine patients had PSA data available. Three out of 3 men (100%) who received it in the first-line for mCRPC achieved a PSA50 response compared to 2 out of 6 men (33%) who received it in the second-line (Fig 2B). Median PFS for first-line abiraterone or enzalutamide for mCRPC was 8.56 months (95% CI: 3.73–9.51 mos.). Median PFS for second-line abiraterone or enzalutamide for mCRPC was 4.59 months (95% CI: 1.83–11.05 mos.) (Table 2).

### Response to immune checkpoint blockade

**Fig 3:**
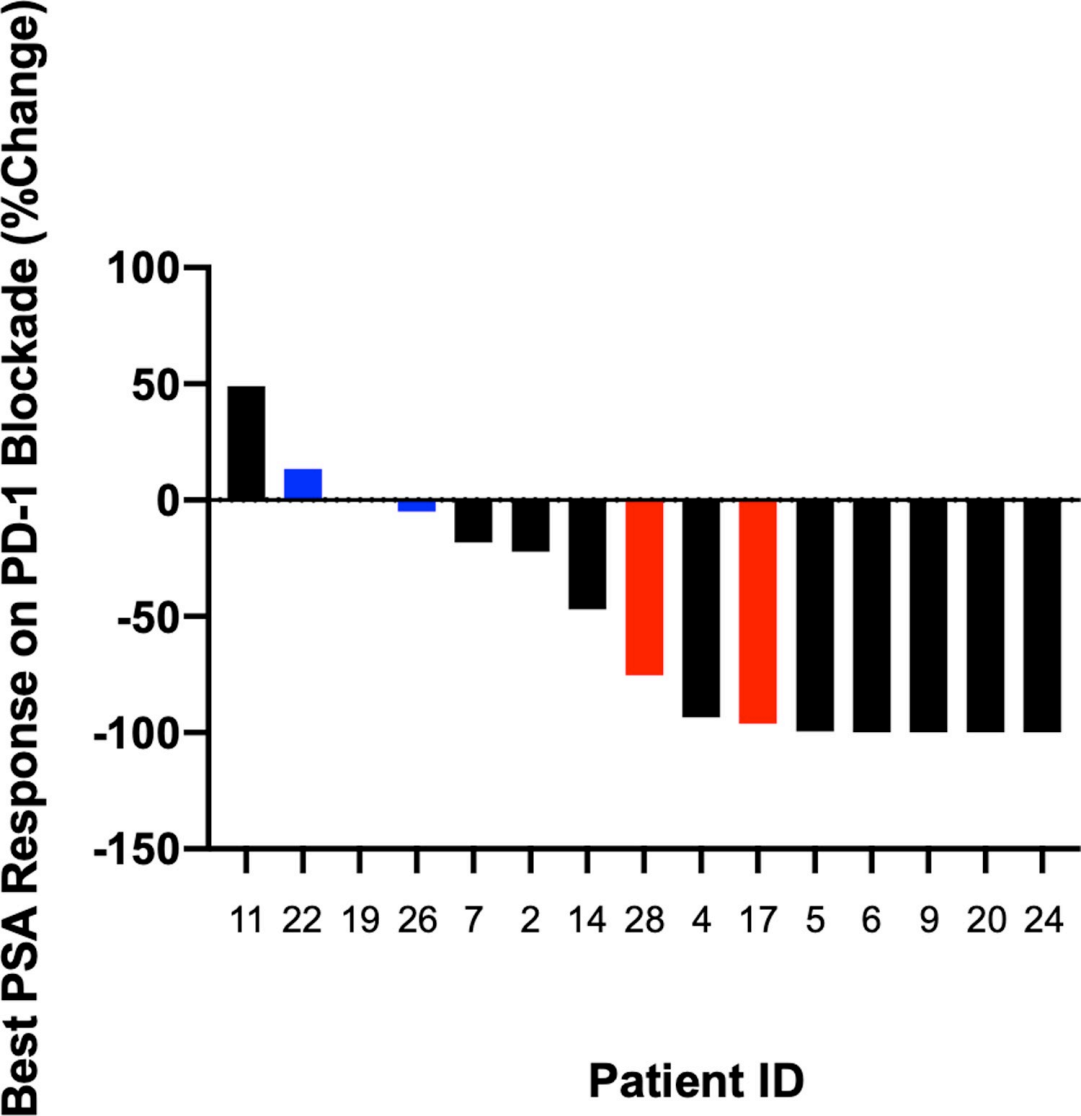
Best PSA response on pembrolizumab in men with MMRd/MSI-H PC. Best % PSA change from baseline following treatment with pembrolizumab. Black = hypermutated (±10 mut/Mb). Blue = not hypermutated. Red = unknown mutational load.

**Fig 4:**
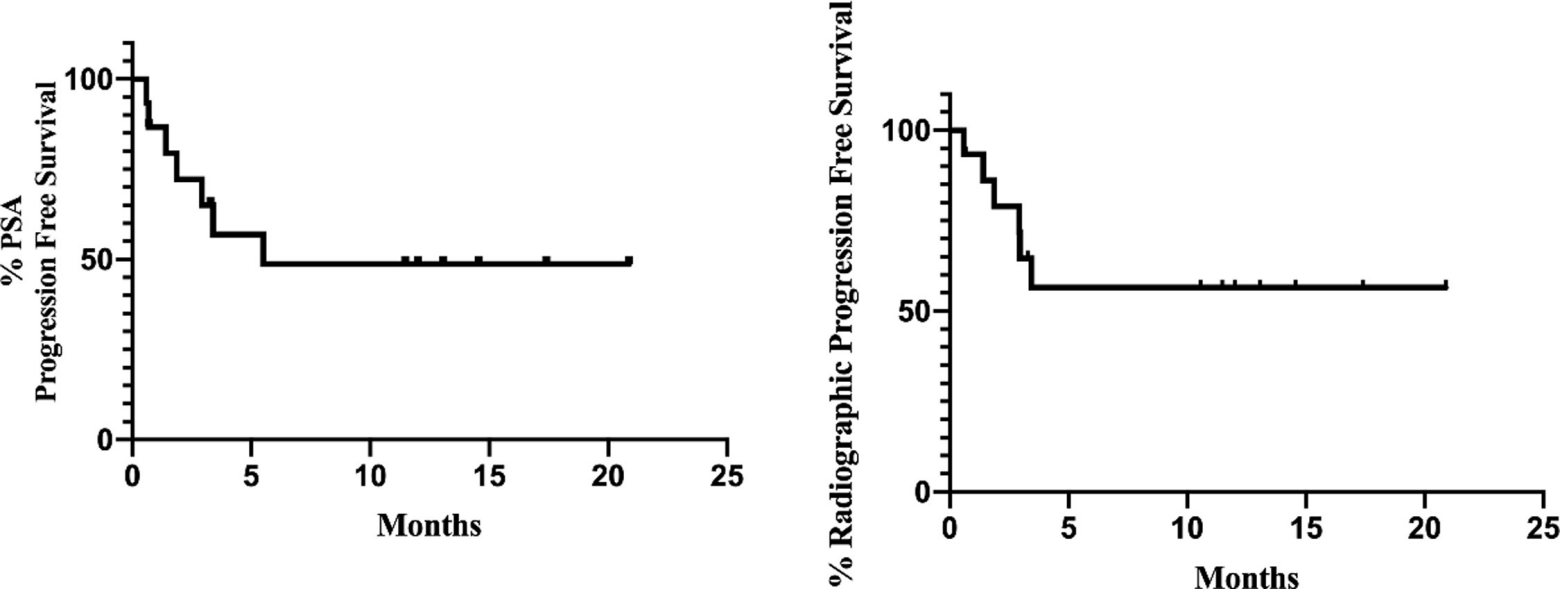
Progression free survival on pembrolizumab in men with MMRd/MSI-H PC. PSA PFS (A) and Radiographic PFS (B).

Seventeen men received pembrolizumab (anti-PD1) monotherapy as standard of care treatment. No one received concurrent radiation therapy. Their molecular and clinical characteristics are shown in Table 3. Two patients were not response evaluable (one patient had limited available treatment data; one discontinued following a single dose due to an immune related adverse event). Eight out of 15 response evaluable patients (53%) had a PSA50 response, including 7 with PSA90 decline (Fig 3). Median radiographic PFS was not reached (95% CI: 1.87-NR mos) and the estimated PFS at 6 months was 64.1% (95% CI: 33.7%-83.4%) (Fig 4A and 4B). Seven of the 8 patients (87.5%) who had a PSA50 response remain on treatment without evidence of progression. The median follow up of these responders is12 months with time on treatment ranging between 3 and 20 months. Of the 8 patients who had a PSA50 response, two had germline sequencing only so did not have tumor mutational load assessed. The remaining 6 all had evidence of hypermutation (i.e. ≥10 mutations/megabase). Of the 7 patients who did not achieve a PSA50 response, 5 (71%) had evidence of hypermutation. There was a trend towards higher response rate to pembrolizumab in patients who had received ≤2 prior therapies vs >2 prior therapies [8/8 (100%) vs. 3/7 (42%), p = .056].

**Table 3:** Molecular and clinical characteristics of patients with MMRd/MSI-H PC who received pembrolizumab. Number of prior therapies includes ADT.

Patient ID	MMR gene mutation	MSI status	Hypermutation Present	Presented with de novo metastatic disease	Number of prior therapies received	Achieved PSA50 response with PD1 inhibitor
2	PMS2	Unknown	Yes	Yes	3	No
3	MSH2	Unknown	Yes	Yes	4	Unknown
4	MSH2	Unknown	Yes	Yes	2	Yes
5	MSH2	Unknown	Yes	No	2	Yes
6	MSH2	Unknown	Yes	Yes	2	Yes
7	MSH2	Unknown	Yes	No	3	No
9	MSH6	Unknown	Yes	No	2	Yes
11	MSH2	Unknown	Yes	No	2	No
14	MSH2	MSI-H	Yes	No	4	No
16	MSH2	MSI-H	Yes	No	2	N/A
17	MSH2	MSI-H	Unknown	No	1	Yes
19	MLH1	MSI-H	Yes	No	1	No
20	MSH2	MSI-H	Yes	No	2	Yes
21	None	MSI-H	No	Yes	2	No
24	PMS2	MSS	Yes	Yes	1	Yes
26	MSH6	MSS	No	Yes	5	No
27	MSH2	Unknown	Unknown	No	1	Yes

## Discussion

There is limited published data on men with MMRd prostate cancer, yet it represents a clinically important and biologically distinct subgroup. To our knowledge, this series represents the largest reported cohort of men with MMRd prostate cancer who have received immune checkpoint blockade. Consistent with prior reports, our patients had evidence of aggressive clinical and pathologic features, with a high incidence of Gleason score ≥8 and high rates of *de novo* metastatic disease (Table 4) [6, 7, 14, 15]. Despite these aggressive features, the men in our cohort fared well on standard hormonal therapies. While formal statistical comparisons are not possible, it is notable that time to castration-resistance, PSA50 response and PFS on first-line abiraterone/enzalutamide were similar to historical controls [16–18]. Interestingly, in our cohort of patients, response to taxane therapy was low in the CRPC setting.

**Table 4:** Comparison of published case series of men with MMRd PC.

	Abida et al.	Antonarakis et al.	Ritch et al.	Graham et al.
	N = 32	N = 13	N = 11	N = 27
GS 8–10, no. (%)	17 (53)	10 (77)	8 (73)	19 (70)
Metastatic Disease at Diagnosis, no. (%)	14 (44)	6 (46)	5 (45)	13 (48)
Presence of Ductal/Intraductal Histology, no. (%)	1 (3)	3 (23)	Not Reported	8 (30)
Presence of Pure Neuroendocrine Histology, no. (%)	3 (9)	1 (8)	Not Reported	0 (0)
Median Time to CRPC, mos.	8.6 (range 1.2–54.2)	55 (95% CI: 50–73)	9.1 (range 5.7–12.6)	14.2 (95% CI: 8.0–32.6)
PSA50 response to 1^st^ line abi/enza for mCRPC, no. (%)	Not Reported	5 (83)	Not Reported	10 (62.5)
Median PFS on 1^st^ line abi/enza for mCRPC, mos.	9.9 (range 3–34.5)	26 (95% CI: 6-NR)	3.9 (0.9–13.0)	8.56 (95% CI: 3.73–9.51)
Received PD-1 blockade, no. (%)	11 (34)	4 (31)	Not Reported	17 (61)
PSA50 response to PD-1 blockade, no. (%)	6 (54.5)	2 (50)	Not Reported	8 (53)
Median PFS on PD-1 blockade, mos.	Not Reported	9 (95% CI: 4–11)	Not Reported	Not Reached (1.87-NR)

Abi, abiraterone; Enza, enzalutamide

Checkpoint inhibitor therapy produced a significant response in a subset of patients with MMRd mCRPC. This data further supports mismatch repair deficiency as a useful biomarker to predict response to immunotherapy. Consistent with prospective studies evaluating immune checkpoint blockade in MMRd cancers, responses are not universal, suggesting that there are other variables that influence efficacy of immunotherapy. Tumor mutational burden (TMB) is another candidate predictive biomarker. However, while MMRd/MSI-H tumors frequently have high TMB, this relationship is imperfect, with up to 30% of MSI-H tumors in one study having a low TMB [19]. Mutational load has been shown to be an independent predictor of response to checkpoint blockade in a variety of solid tumors including melanoma, non-small-cell lung cancer, and urothelial carcinoma [20–22]. In addition the correlation between outcome and tumor mutational burden appears to be linear [23]. Although the majority of our patients had evidence of hypermutation, defined as at least 10 mutations/megabase, exact tumor mutational burden was not reported by most assays, and there may be a threshold that best associates with response [24]. It is also unknown whether the specific MMR gene that is mutated affects response to therapy.

Other factors are likely to affect response to immunotherapy. For example, the tumor microenvironment is affected by therapy and may become more immune suppressive after exposure to cytotoxic chemotherapy [25]. In our data set, receiving pembrolizumab earlier in the disease course was associated with a better response, suggesting that earlier use of immune checkpoint blockade is worthy of further study. However, our limited sample size and the retrospective nature of this analysis limits our ability to definitively conclude that checkpoint inhibitors should be used earlier in the treatment course of mCRPC patients with MMRd.

Prostate cancer with altered MMR machinery has a unique biology with an aggressive phenotype. It represents a unique subset of patients that are more likely to respond to immunotherapy than an unselected group. Further research is needed to predict which MMRd patients will have the best response to immunotherapy and the optimal timing for deploying these drugs in this important patient subset.

## Supporting information

S1 Table Pathologic and genetic characteristics of MMRd/MSI-H prostate cancer cases. (DOCX)

S1 Fig Treatment timelines of individual patients. Included therapies are shown in the legend on the right; in some instances where a patient was receiving ADT they may have been receiving other therapies that are not listed.
(TIFF)
