# Supplementary material for: Mismatch repair deficiency in metastatic prostate cancer: Response to PD-1 blockade and standard therapies
Source: PLoS One. 2020 May 26;15(5):e0233260. doi: 10.1371/journal.pone.0233260 (PMC7250457; doi:10.1371/journal.pone.0233260)
Supplement: S1 Table — (DOCX) [file pone.0233260.s001.docx]

| **Patient ID** | **Institution** | **Variant Histology** | **Gleason Score** | **Tissue source (1=primary, 2=metastatic)** | **Affected MMR gene(s)** | **Germline Mutation** | **Hypermutation present (i.e. >10 mutations/megabase)** | **Microsatellite Instability Status** | **Other Significant Mutations** |
| --- | --- | --- | --- | --- | --- | --- | --- | --- | --- |
| 1 | UMich | Ductal | 8 | 2 | MSH2: one copy loss  MSH6: one copy loss |  | Yes | Unknown | CBLB: one copy loss RB1: one copy loss PRKDC: one copy loss |
| 2 | UMich |  | N/A | 2 | PMS2: p.R315*  PMS2: homozygous copy gain |  | Yes | Unknown | NF1: p.W1314*  TP53: p.Y234C  BRCA2: copy loss  TP53: copy loss BRAF: copy gain  PDGFA: copy gain |
| 3 | UMich |  | 9 | 2 | MSH2: one copy loss |  | Yes | Unknown | RNF43: p.G659fs ASXL1: p.G646fs ERF: p.G299fs and p.G192D |
| 4 | UMich |  | N/A | 2 | MSH2: homozygous deletion |  | Yes | Unknown | PTEN: p.K267fs and p.T131I  NCOR1: p.E529fs and splice donor of exon 16 ASXL1: p.G646fs ERF: p.G299fs ZFHX3: p.Q3197fs AR: Three copies (chrX) |
| 5 | UMich | Ductal | 9 | 2 | MSH2: p.Q348* with LOH |  | Yes | Unknown | AR: p.L702H, p.V716M, and p.S889G NCOR1: Splice donor of exon 34, p.E529fs, and p.S1971fs CHD1: Splice donor of exon 32 ARID2: p.I37fs ACVR2A: p.K437fs ERF: p.S147* KMT2C: p.F2313fs KMT2D: Splice donor of exon 3 |
| 6 | UMich |  | 9 | 2 | MSH2: copy neutral LOH |  | Yes | Unknown | TP53: p.R248Q, copy neutral LOH CTNNB1: p.D32G  ZFHX3: p.R1210* and p.E763fs ASXL1: p.G646fs BAX: p.E41fs HDAC2: p.K11fs, copy neutral LOH HDAC2: copy neutral LOH TP53: copy neutral LOH |
| 7 | UMich |  | 9 | 2 | MSH2: Homozygous deletion (chr2) |  | Yes | Unknown | AR: p.T878A and p.V716M AKT1: p.E17K, activating SPOP: p.F133V RB1: p.T5fs  NCOR1: p.E843fs NCOR1: p.M1675fs ZFHX3: p.Q1378* and p.C1432fs HOXB3: p.P183fs CREBBP: p.D2429fs KMT2D: p.R4904* CHD1: Homozygous deletion (chr2) SPOPL: Homozygous deletion (chr5) |
| 8 | UMich |  | 9 | 2 | MSH2: one copy loss  MSH6: pF1088fs with LOH |  | Yes | Unknown | TP53: p.R248Q SPOP: p.F102C CDH1: p.T229fs with LOH FOXA1: p.A323fs |
| 9 | UMich |  | 9 | 2 | MSH6: p.F1088fs, copy neutral LOH |  | Yes | Unknown | AKT1: p.E17K  MSH6: p.F1088fs TP53: p.R181C and p.R273C ARID1A: p.R693* ATRX: p.K1939fs BAX: p.E41fs EP300: p.L417fs and p.M1470fs RNF43: p.G659fs AR: Amplification (10 copies; chrX) |
| 10 | UMich |  | 9 | 2 | MSH2: homozygous deletion (chr2) |  | Yes | Unknown | AR: p.H875Y APC: p.T1556fs, with LOH ARID1A: p.P2140fs KMT2C: p.K2797fs KMT2C: p.N2842fs KMT2D: p.P2354fs AR: Amplification (chrX) |
| 11 | UMich | Ductal | 9 | 2 | MSH2: p.1828fs with LOH |  | Yes | Unknown | TP53: p.P72H, p.G245S, p.G361fs RNF43: p.G659fs  ETS fusion: TMPRSS2-ETV4 |
| 12 | UW | Ductal | 9 | 1 | MSH2: exon 7 deletion with LOH |  | No | MSS | SPOP: p.F102C  APC: p.N1124Kf2*2  ARID1A: p.G37Afs*14 |
| 13 | UW |  | 9 | 2,1 | MSH2: inversion mutation; breakpoint location chr2: 150445815 and crh2: 47655739 |  | No | MSS | PTEN: copy loss  TP53: p.L252_I254del |
| 14 | UW |  | 9 | 2 | MSH2: p.S676P; one copy loss |  | Yes | MSI-H | AR: p.H865Y*  APC: p.R635M  CDKN1B: one copy loss  BRCA2: one copy loss  SMAD9: one copy loss  FOXO1: one copy loss  RB1: one copy loss |
| 15 | UW |  | 7 | 1 | MSH2: p.Q61X with LOH | Yes | Yes | MSI-H | SPOP: p.Y87C  PTEN loss of function mutations: p.Y346X and p.T319* |
| 16 | UW | Ductal | 7 | 1 | MSH2: (chr2) GRHL2 (chr8) fusion |  | Yes | MSI-H | None |
| 17 | UW |  | 9 | 1 | MSH2: c.942+3A>T  Note: MSH6 loss of expression by IHC | Yes | Unknown | MSI-H | None |
| 18 | UW | Ductal | 7 | 1 | MSH6: p.L634Efs*5 |  | Yes | MSS | PTEN loss of function mutations: c.917_918del, c.968dup, c.302TtoC |
| 19 | UW | Ductal | 7 | 2 | MLH1: Focal deletion of 145kb on chromosome 3 which includes the last exon of MLH1 (breakpoints chr3: g.37091968 and chr3: g.37237643) |  | Yes | MSI-H | FOXA1 rearrangement  AR: p.W742L  PIK3CA: p.H1047R  ETS fusion: TMPRSS2-ERG |
| 20 | UW |  | 8 | 1 | MSH2: translocation involving intron 15 of the MSH2 gene (chr2) with CLK4 gene (chr5); approximate breakpoint chr2: g.47707049-chr5:g.178038044 |  | Yes | MSI-H | PTEN, EPHA5, EPHB6, GLI1, SMARCB1: Loss of function mutations  ARID1A, JAK1, PIK3R1, and TSC2: Missense mutations |
| 21 | UW |  | 9 | 2 | None |  | No | MSI-H | None |
| 22 | UW |  | 9 | 2 | MSH2: Inversion chr2 (approximate breakpoints chr2:4688726 and chr2: 47662135) |  | Yes | MSI-H | PTEN: loss of function frameshift mutations  AR: p.T878A |
| 23 | UW | Ductal | 9 | 1 | MSH2: Inversion chr2 with a breakpoint in intron 7 of MSH2 (chr2: g.47647684-49777102inv), with LOH |  | Yes | MSI-H | AR: p.H875Y and p.V716M |
| 24 | UW |  | 7 | 2 | PMS2: c.989-1G>T |  | Yes | MSS | AR: p.W742C |
| 25 | UW |  | N/A | 2 | MSH2: homozygous deletion |  | Yes | MSI-H | AR: p.T878A |
| 26 | UW |  | 9 | 2,1 | MSH6: p.C1269Mfs*6 |  | No | MSS | FOXA1: p.F266Kfs*11  CHD1: bi-allelic copy loss  AR amplification  APC: p.N1531Kfs*2 |
| 27 | UW |  | 9 | N/A- germline only | MSH2: deletion exons 1-6 | Yes | Unknown | Unknown | Unknown |
