# Supplementary figures and images for: Mismatch repair deficiency in metastatic prostate cancer: Response to PD-1 blockade and standard therapies
Source: PLoS One. 2020 May 26;15(5):e0233260. doi: 10.1371/journal.pone.0233260 (PMC7250457; doi:10.1371/journal.pone.0233260)

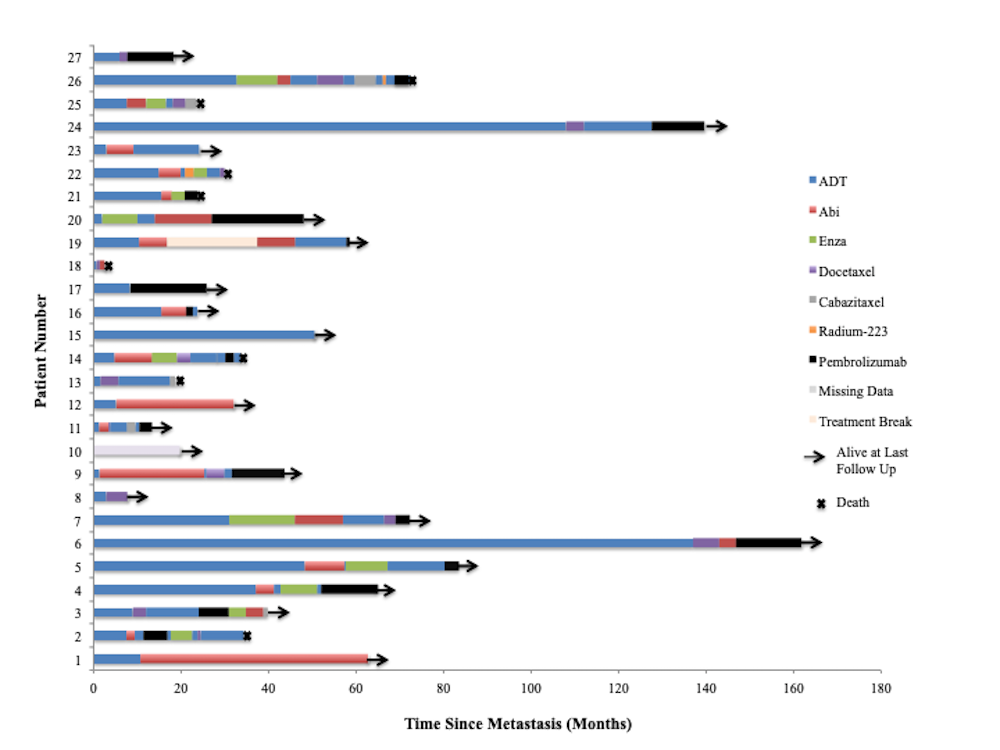

Supplement: S1 Fig — Included therapies are shown in the legend on the right; in some instances where a patient was receiving ADT they may have been receiving other therapies that are not listed. (TIFF) [file pone.0233260.s002.tiff]
